# 16S rRNA gene amplicon sequences from coastal sediments at the Sonoran Desert and Gulf of California interface

**DOI:** 10.1128/mra.01003-25

**Published:** 2025-10-14

**Authors:** Cristal Ramos-Madrigal, Esperanza Martínez-Romero, Yunuen Tapia-Torres, Luis E. Servín-Garcidueñas

**Affiliations:** 1 Laboratorio de Microbiómica, Escuela Nacional de Estudios Superiores Unidad Morelia, UNAM 7180, Morelia, Michoacán, Mexico; 2 Posgrado en Ciencias Biológicas, Unidad de Posgrado, Ciudad Universitaria, Ciudad de México, Mexico; 3 Programa de Ecología Genómica, Centro de Ciencias Genómicas, UNAM 7180, Cuernavaca, Morelos, Mexico; Portland State University, Portland, Oregon, USA

**Keywords:** halophiles, microbial diversity, extreme environments, Sonoran desert, salt crusts, sand sediments, archaea, bacteria, hypersaline ecosystems, extremophiles

## Abstract

We report the taxonomic composition of bacterial and archaeal communities from sand and salt-crust sediments in the coastal Sonoran Desert, Mexico. Analysis of 16S rRNA gene amplicon sequences revealed Halobacterota, Bacteroidota, and Proteobacteria as dominant phyla, providing a resource to explore microbial diversity in extreme environments.

## ANNOUNCEMENT

In the Sonoran Desert along the Gulf of California, extensive salt crusts and sediments form under hypersaline conditions, temperature extremes, and intense solar irradiance. Here, we profiled bacterial and archaeal diversity in these coastal habitats using 16S rRNA amplicon sequencing to provide a high-resolution taxonomic profile of desert sediments.

Samples of salt crust and surface sand were collected in March 2024 at a coastal site (31°42′36″N, 113°58′07″W). Samples were stored in sterile bags at room temperature until DNA extraction. DNA was extracted with the DNeasy PowerSoil Pro kit (Qiagen, Germantown, MD, USA), following the manufacturer’s instructions. DNA was quantified with the QuantiFluor dsDNA System (Promega, Madison, WI, USA). The salt-crust and sand samples were analyzed with two primer sets targeting different 16S rRNA regions. The V3-V4 region was amplified using the primers 341F (5′-CCTACGGGNGGCWGCAG-3′) and 805R (5′-GACTACHVGGGTATCTAATCC-3′), hereafter “Salt Crust 1” and “Sand” (1). The V5-V6 region was amplified using the archaeal-biased primers 787F (5′-GGATTAGATACCCSBGTAGT-3′) and 1059R (5′-ACGGGCGGTGTGTACAAG-3′), hereafter “Salt Crust 2” (2). The sand sample amplified only with primers 341F/805R. PCR was performed with an initial denaturation at 95°C for 3 min; 25 cycles of 95°C for 30 s, 55°C for 30 s, and 72°C for 30 s; a final extension at 72°C for 5 min. Libraries were prepared with the Nextera XT Index Kit (Illumina), following the manufacturer’s instructions. Libraries were sequenced on an Illumina MiSeq (2 × 301 bp; Macrogen Co., Seoul, South Korea). Read quality was assessed with FastQC v0.11.8 (with default settings) (3) and trimmed at Q20 using Trimmomatic v0.39 (4). Reads were further processed in QIIME 2 v2024.10 (5, 6) with q2-dada2 (7) for denoising and chimera removal. Taxonomy was assigned to amplicon sequence variants (ASVs) using the q2-feature-classifier (classify-consensus-vsearch method) (8) with the SILVA v138 SSURef Nr99 database (9).

**Fig 1:**
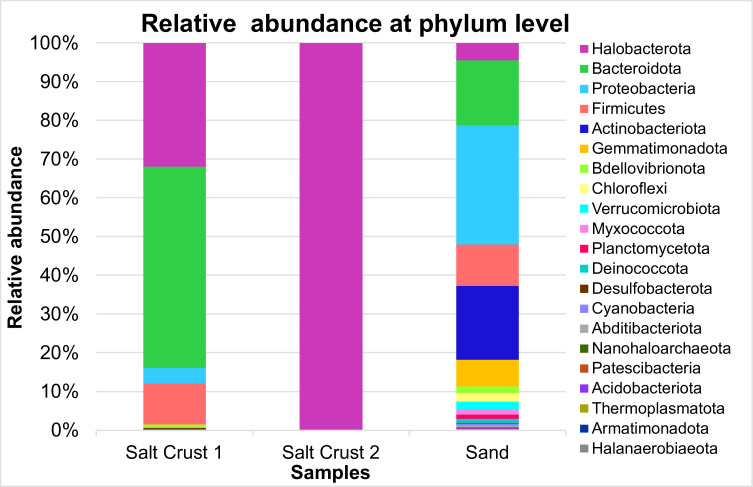
Relative abundance of bacterial and archaeal phyla based on 16S rRNA amplicon sequencing. Sand and Salt Crust 1 were amplified with 341F/805R primers for the detection of Bacteria and Archaea. Salt crust 2 used archaeal-biased 787F/1059R primers to further investigate archaeal diversity.

Sand showed the highest number of unique ASVs, whereas salt crust yielded fewer but more specialized lineages, consistent with hypersaline conditions and primer specificity (Table 1). In the sand sample, the dominant phyla were Proteobacteria (30.70%), Actinobacteriota (19.08%), and Bacteroidota (16.83%). In Salt Crust 1, the most abundant phyla were Bacteroidota 51.95%, Halobacterota 31.96%, and Firmicutes 10.56%. In Salt Crust 2, 99.92% of sequences belonged to Halobacterota, whereas 0.07% corresponded to Actinobacteriota (Fig. 1). Thus, different primer sets revealed complementary diversity. Salt Crust 1 and Salt Crust 2 shared 13 archaeal genera; they shared seven and five genera, respectively, with the Sand sample. Two genera were exclusive to Salt Crust 1, and four were exclusive to Salt Crust 2, indicating complementary detection of archaeal groups.

**TABLE 1:** Description of summary data obtained from the sediment samples in the study

Primers	Sample	Relative abundance of Bacteria (%)	Relative abundance of Archaea (%)	Total paired-end reads	Reads after quality filtering (Q20)	Reads after denoising and chimera removal	No. of ASVs	Identified genera
341F 805R	Salt Crust 1	67.74	32.25	181,801	135,455	52,202	271	36
	Sand	95.32	4.67	91,715	48,328	33,797	789	125
787F 1059R	Salt Crust 2	0.07	99.92	87,922	66,228	20,883	110	17

Halophiles, including *Natronorubrum tibetense* (identified in all samples), *Natronococcus occultus* (Salt Crusts 1 and 2), *Halalkalicoccus jeotgali* (Salt Crust 2), *Natrinema pellirubrum* (Salt Crust 1), and marine halophilic bacteria of the order *Oceanospirillales* (Salt Crust 1 and Sand), have been reported in the Sonoran Desert and other hypersaline environments (10–16). Halophiles have biotechnological potential (17–19). These findings highlight the coastal zone of the Sonoran Desert as a microbial reservoir and a priority for further investigation.

## Data Availability

Raw sequences are available in the NCBI Sequence Read Archive (SRA) under accession numbers SRX28081615, SRR32725451, and SRR32725450, within BioProject PRJNA1236701.
